# Surveying the potential of secreted antimicrobial peptides to enhance plant disease resistance

**DOI:** 10.3389/fpls.2015.00900

**Published:** 2015-10-27

**Authors:** Susan Breen, Peter S. Solomon, Frank Bedon, Delphine Vincent

**Affiliations:** ^1^Plant Sciences Division, Research School of Biology, The Australian National UniversityCanberra, ACT, Australia; ^2^Department of Economic Development, AgriBioBundoora, VIC, Australia; ^3^AgriBio, La Trobe UniversityBundoora, VIC, Australia

**Keywords:** transgenic plants, mass spectrometry, plant-microbe interaction, pathogen resistance, ribosomal and nonribosomal antimicrobial peptides, immunity

## Abstract

Antimicrobial peptides (AMPs) are natural products found across diverse taxa as part of the innate immune system against pathogen attacks. Some AMPs are synthesized through the canonical gene expression machinery and are called ribosomal AMPs. Other AMPs are assembled by modular enzymes generating nonribosomal AMPs and harbor unusual structural diversity. Plants synthesize an array of AMPs, yet are still subject to many pathogen invasions. Crop breeding programs struggle to release new cultivars in which complete disease resistance is achieved, and usually such resistance becomes quickly overcome by the targeted pathogens which have a shorter generation time. AMPs could offer a solution by exploring not only plant-derived AMPs, related or unrelated to the crop of interest, but also non-plant AMPs produced by bacteria, fungi, oomycetes or animals. This review highlights some promising candidates within the plant kingdom and elsewhere, and offers some perspectives on how to identify and validate their bioactivities. Technological advances, particularly in mass spectrometry (MS) and nuclear magnetic resonance (NMR), have been instrumental in identifying and elucidating the structure of novel AMPs, especially nonribosomal peptides which cannot be identified through genomics approaches. The majority of non-plant AMPs showing potential for plant disease immunity are often tested using *in vitro* assays. The greatest challenge remains the functional validation of candidate AMPs in plants through transgenic experiments, particularly introducing nonribosomal AMPs into crops.

## Introduction

Peptides with antimicrobial activities, or antimicrobial peptides (AMPs), have emerged as key components of the innate immune system in almost all living organisms since their first discovery from culture supernatant of the soil bacteria *Bacillus brevis* more than seven decades ago (Dubos, 1939a,b). AMPs are naturally synthesized low molecular mass products [up to 100 amino acids (AAs)] that act against microbial pathogens. AMPs are structurally and biochemically highly diverse but typically include positively charged AAs and hydrophobic or hydrophilic moieties facilitating more or less their aqueous solubility and interaction with the negatively charged parts of the phospholipidic microbial cell membranes (reviewed in Montesinos, 2007; Mousa and Raizada, 2015; Wang et al., 2015). As a consequence of their diversity, specific AMPs are more effective in interacting and disrupting targeted microbial membranes, such as fungal pathogens for example (Marx, 2004; Hegedüs and Marx, 2013; Vincent and Bedon, 2013; van der Weerden et al., 2013).

AMPs can be classified as either ribosomal or nonribosomal according to their mode of synthesis by the cells. Ribosomal peptides are gene-encoded peptides usually resulting from cleavage of a pro-protein, while nonribosomal peptides are assembled by multimodular enzymes called NonRibosomal Peptide Synthetases (NRPS). These NRPSs are usually organized in one operon, for bacteria, or in gene clusters for eukaryotes, and they synthesize one peptide per gene cluster or operon. NPRSs can generate macrocyclic peptides with an unusual structural diversity achieved through the assembly of not only the 20 canonical AAs, but also D-configured- and β-AAs, methylated, glycosylated and phosphorylated residues, heterocyclic elements and even fatty acid (FA) chains (Marahiel, 2009). Cyclic lipopeptides containing FAs are examples of such nonribosomal peptides (for review Lee and Kim, 2015; Patel et al., 2015). Ribosomal peptides are difficult to predict *in silico* from transcriptomic and genomic sequencing projects due to their small size and high diversity. There are also no known generic cleavage sites that could indicate a potential peptide. Tools are available allowing *in silico* genome mining, as was demonstrated for *Bacillus sp*. (Aleti et al., 2015). The most comprehensive AMPs database to date, named ADAM, is publically available and currently contains 7007 unique peptide sequences and 759 structures (http://bioinformatics.cs.ntou.edu.tw/ADAM/links.html) (Lee et al., 2015), yet some of the nonribosomal AMPs listed in this review are missing. Other AMP databases exist (reviewed in Holaskova et al., 2015). Methods other than genomics are therefore advantageous, particularly mass spectra (MS)-based proteomics strategies as they allow the direct identification of the AMPs and their isoforms. MS analysis of secreted peptides generates reliable and useful information such as the molecular weight, the AA sequence, as well as the length of the FA chain in the case of lipopeptides (Zhao et al., 2014). Furthermore, MS imaging technology has emerged as a powerful tool to not only identify novel AMPs but also locate them *in situ* (Debois et al., 2013). Nuclear magnetic resonance (NMR) analyses are complementary because they yield structural elucidation such as cyclic structures (Wäspi et al., 1998; Sammer et al., 2009).

**Figure 1:**
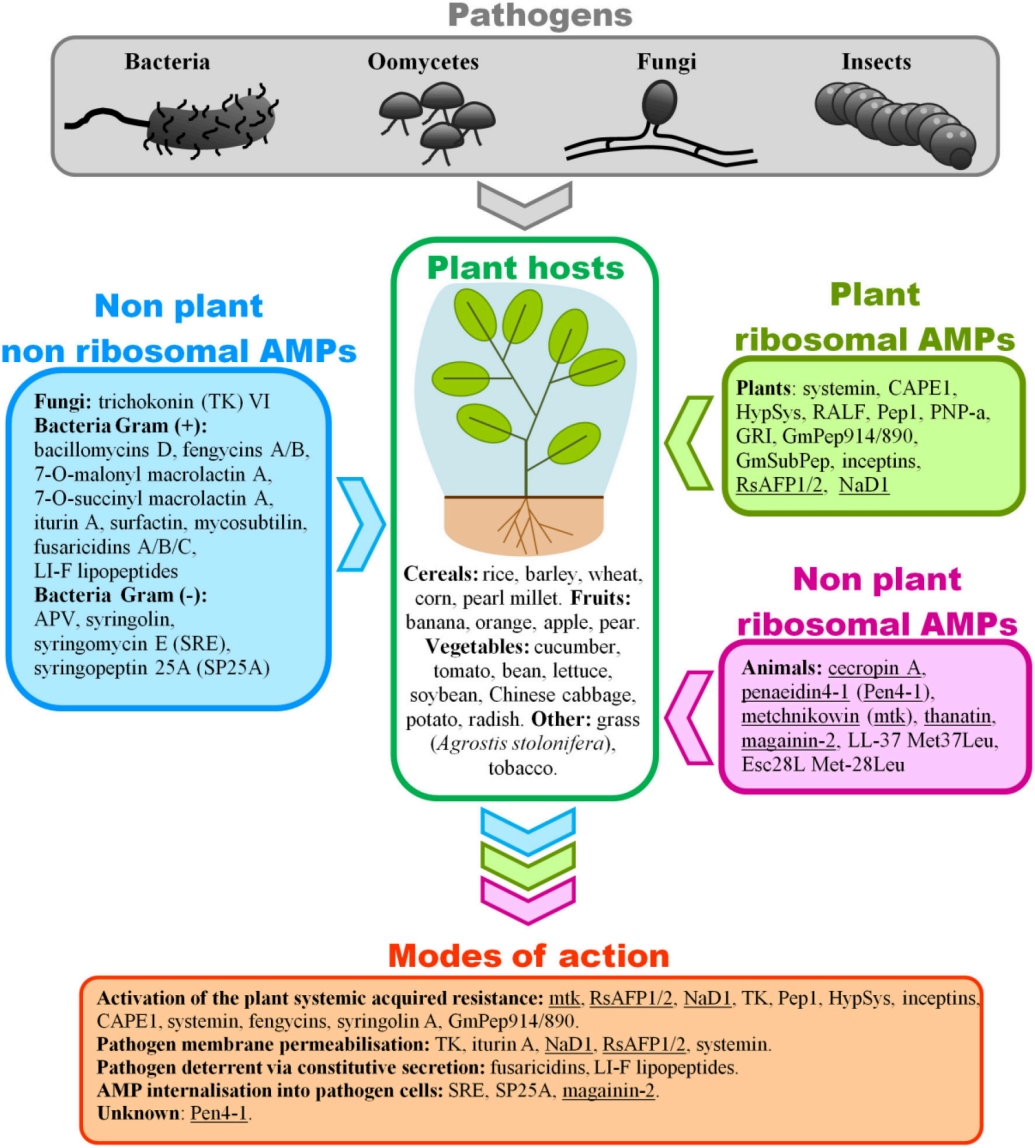
**Summary of the modes of action of non-plant and plant related AMPs involved in host resistance against plant pathogens reviewed here**. A number of pests (pathogens and herbivores) can attack a crop which deploys an arsenal of defense to stay healthy. Yet some pests manage to break through plant defense mechanisms. Ribosomal and non-ribosomal AMPs synthesized by organisms other than the crop of interest offer a way to increase the plant resistance levels against pests. Their mode-of-actions, where known, mainly boosts systemic acquired resistance (SAR) of the plant host, particularly through the induction of pathogenesis-related (PR) proteins as reported for mtk, TK, CAPE1, Pep1, as well as the induction of reactive oxygen species (ROS) as reported for RsAFPs, NaD1, TK, Pep1, HypSys. Another manifestation of SAR implicates phytohormones signaling pathways as well as secondary metabolites. A number of AMPs have been associated with salicilic acid (SA) as reported for TK, inceptins, and CAPE1, jasmonic acid (JA) as reported for inceptins, CAPE1, Pep1, and systemin, and ethylene as reported for Pep1. Fengycins have been implicated in the production of phenolic compounds derived from the plant defense-related phenylpropanoid metabolism, while GmPep914/890 enhanced phytoalexin production. Syringolin A was shown to induce the transcription of *Pir7b, Pir2, Pir2, and Rir1* defense genes. Another common mode of action in plant defense mechanism operates by compromising the cell membrane permeability of the pathogen. Indeed TK was involved in the change of fungal membrane permeability and disintegration of subcellular structures, while iturin A was associated with the creation of transmembrane channels thus resulting in the release of vital ions such as K+. NaD1, interacting with PIP2, triggered the granulation of the hyphal cytoplasm followed by cell death. The interaction of RsAFP1/2 with fungal membrane glucosylceramide (GlcCer) resulted in the activation of MAP kinase and cell wall integrity signaling pathways. Systemin caused a rapid alkalinization of the extracellular space via blockage of a proton pump in the cell membrane. AMP internalization into pathogen cells can be mediated by cell wall degrading enzymes (CWDEs) as reported for syringomycin E (SRE), syringopeptin 25A (SP25A), and magainin-2. Finally some AMPs act as pathogen deterrent via constitutive secretion as demonstrated for fusaricidins, and LI-F lipopeptides. (1) AMPs used for transgenic experiments in plants are underlined. (2) Pathogens listed are bacteria *(Erwinia amylovora, Pseudomonas syringae pathovars, P. aeruginosa, Serratia marcescens, Bacillus subtilis, Pectobacterium carotovorum, Ralstonia solanacearum, Dickeya dadantii), oomycetes (Pythium irregular, P. dissotocum, P. ultimum, P. aphanidermatum, Phytophthora infestans, P. parasitica, P. nicotianae), fungi (Cochliobolis heterostrophus, Colletotrichum graminicola, C. gloeosporioides, C. higginsianum, Rhizoctonia cerealis, R. solani, Fusarium oxysporum pathovars, F. graminearum, F. culmorum, F. verticillioides, Verticillium dahliae, Blumeria graminis, Botrytis cinerea, Penicillium expansum, P. crustosum, Rhodotorula pilimanae, Rhizopus sp., Alternaria citri, Botryosphaeria sp., Fusicoccum aromaticum, Lasiodiplodia theobromae, Sphaerotheca fuliginea, Bremia lactucae, Cladosporium cucumerinum, Ascochyta citrullina, Sclerotinia homoecarpa, Magnaporthe oryzae, Aspergillus flavus, Sclerospora graminicola), and insects (Spodoptera litura, S. frugiperda, grasshopper)*.

**Figure 2:**
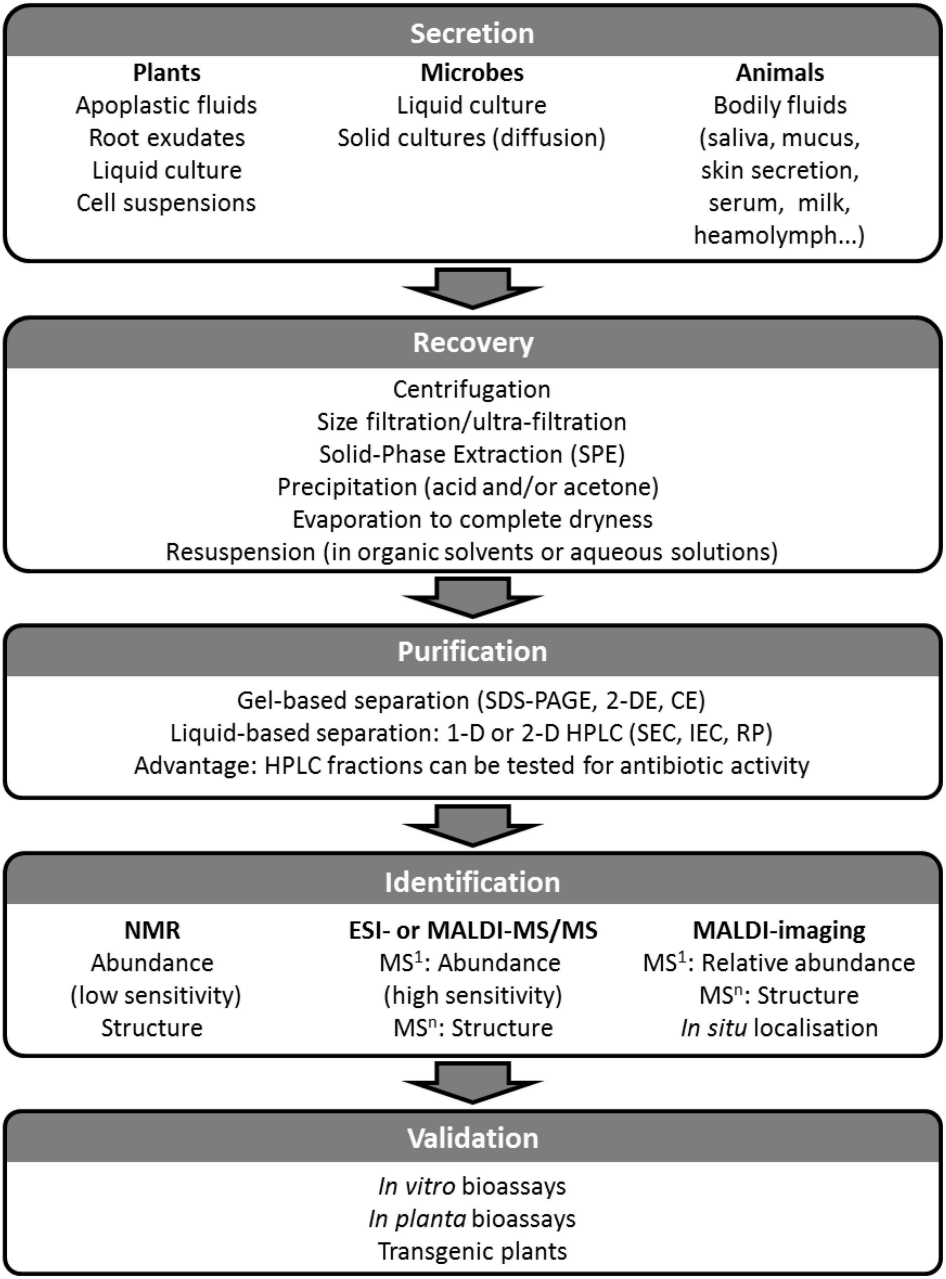
**Diagram for the identification and validation of AMPs bearing biocontrol potential in plant immunity**. Secreted AMPs can be recovered from any inter- or extra-cellular space and from any organism. Being dilute, secreted AMPs will need to be concentrated using one or a combination of the methods listed in the Recovery box; prior to their purification AMPs must be solubilized. Purification involves separation steps either using gel-based or gel-free techniques. Purified AMPs can then be analyzed using NMR and/or MS which will yield identification and structural elucidation. The final step in the process is the validation of the novel AMP as a biocontrol agent using bioassays, and ultimately introducing it into the crop by transgenesis to assess the level of pest resistance it confers.

Plant protection and resistance against pathogens have been traditionally and are still currently addressed with chemical uses and breeding programs. Nevertheless, the possibilities arising from the study of AMPs will contribute to control the plant pathogens whose virulence is driven by perpetual adaptation through mutation. The aim of this review is to discuss naturally synthesized and secreted AMPs offering biocontrol potential for crop species. In this review, we chose to focus on well-studied AMPs representative of the main taxa such as bacteria, fungi, animals, and plants. The first section covers the plant-secreted peptides induced as a defense mechanism following pathogen attack or herbivory. The last section focuses on peptides secreted by non-plant species (bacteria, fungi and animals) that are subject to microbial challenges, and the potential of these peptides to help plant species fight fungal diseases by applying a biocontrol approach. Figure 1 illustrates AMPs described in the review with their mode-of-action where known. Figure 2 outlines a workflow to isolate, identify and validate AMPs bearing biocontrol potential in plant immunity. We discuss successful cases of increased pathogen resistance in transgenic plants and their potential for increased crop yield. Additional information on transgenic plants expressing AMPs toward improved disease resistance can be found in the reviews from Ramadevi et al. (2011) and Holaskova et al. (2015). Not covered in this review is a promising strategy for synthetic peptides production through combinatorial chemistry which offers an alternative approach to developing AMPs for agricultural applications (López-García et al., 2002; Choi and Moon, 2009; Rebollar et al., 2014).

## Secreted plant ribosomal peptides for signaling and defense

The advances in sequencing technology over the last few decades have led to an increasing number of plant genomes being widely available. The first sequenced plant genome was *Arabidopsis thaliana* (Intitative, 2000). Analysis of this genome revealed that plants encode many more predicted peptide transporters and receptors than was expected based on animal systems (Intitative, 2000; Shiu and Bleecker, 2003). This implies that many of the plant peptides involved in plant growth and development, cell-to-cell communication and defense responses have yet to be identified. Plant AMPs are also a rich source of plant defense compounds which can be grouped based on their structure. The 8 main classes in plant AMPs are cyclotides, lipid transfer proteins, defensins, thionins, snakins, hevein-like, vicillin-like, and knottins (Goyal and Mattoo, 2014). This section will focus on known plant endogenous peptides which are secreted upon recognition of microbial colonization of the plant and aid in plant defense signaling. Some of these have been comprehensively reviewed (Ryan and Pearce, 2003; Boller, 2005; Farrokhi et al., 2008; Yamaguchi and Huffaker, 2011) but updated information will be presented here. The defensins will be looked at in detail as they have been successfully taken through to field trials recently. Other AMPs showing induction of resistance in transgenic plants have also been described previously (Oard and Enright, 2006; Muramoto et al., 2012; Verma et al., 2012; Zhu et al., 2012). These other classes of AMPs with increased resistance have been well reviewed (Goyal and Mattoo, 2014). Where possible the receptors detecting these endogenous peptides will also be discussed. Table 1 summarizes the plant secreted peptides discussed in this review; AMP precursors and receptors, where known, are indicated.

**Table 1:** **Plant ribosomal AMPs**.

**Peptide name**	**AA length**	**Precursor protein**	**Origin species**	**Target pests**	**Peptide receptor**	**References**
Systemin	18	Prosystemin	Tomato, Potato, Pepper, and Nightshade	Wounding insects	BRI1 binds systemin	Pearce et al., 1991
					Another unidentified receptor initiates signaling	
HypSys	15–20	preproHypSys	Tobacco, Tomato, Petunia, Sweet potato, Black nightshade, Potato	Wounding insects	Not known	Pearce et al., 2001
Pep1	23	PROPEP1	*A. thaliana*, Tomato, Potato, Maize	*Pythium irregular; Pythium dissotocum*	PEPR1 and PEPR2	Huffaker et al., 2006
GmPep914	8	GmPROPEP914	Soybean	Not known	Not known	Yamaguchi et al., 2011
GmPep890	8	GmPROPEP890	Soybean	Not known	Not known	Yamaguchi et al., 2011
GmSubPep	12	Glyma18g48580	Soybean	Not known	Not known	Pearce et al., 2010
CAPE1	11	PR-1b (P14a)	Tomato	Bacteria and insects	Not known	Chen et al., 2014
RsAFP1	44	RsAFP1	Radish	Pathogenic fungi and yeast	Not known	Terras et al., 1992
RsAFP2	36	RsAFP2	Radish	Pathogenic fungi and yeast	Not known	Terras et al., 1992
NaD1	47	NaD1 precursor	Ornamental tobacco (*Nicotiana alata*)	Pathogenic fungi	Not known	van der Weerden et al., 2008
Inceptins	Nov-13	Chloroplastic ATP synthase γ-subunit	Cowpea (*Vigna unguiculata*)	Wounding insects	Not known	Schmelz et al., 2006

### Plant signaling peptides

#### Systemin and its receptor: The first peptide acknowledged as a plant hormone

Systemin is the most well-known secreted plant peptide involved in activation of defense signaling and it was the first peptide to be acknowledged as a plant hormone (Ryan and Pearce, 2003). Systemin is an 18-AA peptide which is derived from a 200-AA precursor protein called prosystemin (Pearce et al., 1991; McGurl and Ryan, 1992; Farrokhi et al., 2008; Table 1). The isolation of systemin was achieved by feeding cut *Solanum lycopersicum* (tomato) stems with a few microliters of tomato leaf juice, causing the plants to produce proteinase inhibitor proteins, including systemin (Pearce et al., 1991; Ryan and Pearce, 1998). Prosystemin localizes in the cytoplasm of phloem parenchyma cells, however upon wounding or death of these cells, prosystemin is processed to systemin, probably by proteinases. This allows systemin to diffuse into the apoplast and be detected by receptors on the plasma membrane of mesophyll cells (Boller, 2005). Systemin induces protease inhibitor production within local tissue in order to suppress wounding by insect proteases. However, it also leads to a systemic wound response by activating the jasmonic acid (JA) signaling pathway (Stratmann, 2003; Farrokhi et al., 2008). The production of this peptide appears to be under diversifying selection which suggests that some herbivores may have evolved defenses against systemin signaling (Boller, 2005). Prosystemin has only been found in species of the Solaneae a subtribe of the Solanaceae family e.g., tomato, potato, pepper and nightshade but not in tobacco, another Solanaceae (Scheer and Ryan, 2002; Ryan and Pearce, 2003). Since systemin has not been identified in tobacco this plant also shows no alkalization response to systemin treatment (0.0025–25 nM) (Ryan and Pearce, 2003). Therefore, it seems systemin is restricted to a few species and is not found globally in plants. This suggests that for the Solaneae plants, pre-treatment with systemin could reduce the wounding effects of insects in the field. Systemin pre-treatment also has the potential to reduce other infections, for example virus and bacterial infections transmitted by insects.

Over a decade after the first report of systemin its tomato cell surface receptor was identified (Scheer and Ryan, 2002). SlSR160 is a 160 kDa receptor belonging to the family of leucine rich repeat receptor-like kinases (LRR-RLKs), and like classical LRR-RLKs, it contains an extracellular leucine rich repeat domain, a transmembrane domain and an intracellular kinase domain. In cell culture experiments, systemin detection by SlSR160 activates a complex signaling pathway which results in activation of a mitogen-activated protein kinase (MAPK), rapid alkalinization of the extracellular medium via blockage of a proton pump in the cell membrane, the activation of phospholipases along with activation of phytodienoic acid and jasmonic acid (JA) for defense signaling (Felix and Boller, 1995; Schaller and Oecking, 1999; Ryan and Pearce, 2003). It was later discovered that the systemin receptor, SlSR160, and the brassinosteroid receptor from *A. thaliana*, AtBRI1, are homologs which contain all the same domains, the most conserved of which have 83–90% similarity (Montoya, 2002; Scheer and Ryan, 2002). BRI1 is the cell surface receptor for the brassinosteroid pathway which detects the hormone brassinolide (BL), a regulator of plant growth and development. This shows dual function for this receptor in both plant defense and maintenance of growth and development. In recent years however, the dual function of BRI1/SR160 has come into question. Initially dual function was demonstrated by the expression of tomato SR160/BRI1 in tobacco which allowed the transgenic plants to respond to systemin treatment (Scheer et al., 2003). A mutant tomato *Slbri1* gene called *cu-3*, was used to further examine the dual function of SR160/BRI1. The tomato *cu-3* plants showed a Brassinosteroid (BR) deficient growth phenotype i.e., stunted growth and curled leaves, however these plants also could not induce systemin signaling (Scheer et al., 2003). Confirming this receptor has a dual role in plant defense as well as in growth and development (Montoya, 2002; Scheer et al., 2003). However, more recently it was shown that the reduced responsiveness of *Slcu3* plants to systemin was due to the stunted nature of these plants and *Slcu3* cell cultures responded the same as the wild type (WT) to treatment with systemin (Holton et al., 2007). These results have led to some controversies in this field and a more recent study has sought to try and understand these conflicting results. Malinowski et al. (2009) showed by expression of tomato BRI1 in tobacco cells that BRI1 binds to systemin however the systemin signaling did not show any increase by the overexpression of tomato BRI1. This report confirms results previously described by Holton et al. (2007), that silencing of SlBRI1 in tomato results in a *bri1* phenotype but did not affect systemin response (Malinowski et al., 2009). These results imply that BRI1 can bind systemin but it is not the ligand that initiates intracellular signaling (Malinowski et al., 2009). Therefore, there could be another ligand for systemin yet to be identified. The newest data also explains why *A. thaliana* which contains BRI1 cannot sense systemin treatment or activate downstream defense signals. In *A. thaliana* there is some redundancy within the BRI1 gene family as three other cell surface receptors have been identified with high sequence similarity, BRI1-like (BRL1, BRL2, and BRL3). It was shown that BRL1 and BRL3 can complement BRI1 but BRL2 cannot, BRL2 is required for provascular differentiation in leaves (Clay and Nelson, 2002; Caño-Delgado et al., 2004). Given there is a gene family in *A. thaliana*, it is highly likely that this is true in Solanacea species. Therefore, it could be that one of these other receptors is required for systemin signal transduction.

#### HypSys: The first peptides identified in tobacco

HypSys's are another group of well-known wound-induced peptides which show functional relatedness to systemin and contain multiple hydroxyproline residues. These polypeptide hormones were first identified in tobacco using cell suspension assays treated with a crude peptide fraction from tobacco leaves which resulted in the alkalinization of medium. Two 18-AA polypeptide hormones were identified in tobacco and found to be derived from each end of a 165-AA polyprotein hormone precursor, pro-TobSys-A (Pearce et al., 2001; Table 1). These two peptides were named tobacco hydroxyproline-rich systemin (TobHypSys) I and II (Ryan and Pearce, 2003). Although HypSys's may have similar downstream functions to systemin, it appears the physical properties and the processing of these two peptides differ greatly. The synthesis of HypSys potentially involves the secretory pathway given the presence of carbohydrate residues and hydroxyproline residues, while in contrast systemin contains no hydroxyprolines and no carbohydrate residues (Pearce et al., 2001). HypSys's have also been identified in a wider variety of plants than systemin including tomato (TomHypSys I, II and II), petunia (PhHypSys I, II, and III) sweet potato, black nightshade and potato (Pearce and Ryan, 2003; Pearce et al., 2007, 2009; Chen et al., 2008; Bhattacharya et al., 2013). The TomHypSys are 20, 18, and 15-AAs in length, respectively, while the PhHypSys I, II, and III are 19, 20, and 18-AAs in length, respectively (Pearce and Ryan, 2003; Pearce et al., 2007). In these identified precursor proteins for the HypSys peptides it was noted that a 30 base pair (bp) sequence around the peptidase splice site was conserved in all, this conservation and the high sequence homology over the whole protein is allowing faster and easier identification of these peptides therefore avoiding long troublesome purification from leaf extracts (Bhattacharya et al., 2013).

The 146-AA precursor protein of the three TomHypSys peptides localizes to the cell wall matrix in the phloem parenchyma cells of tomato leaves (Narváez-Vásquez et al., 2005). The tomato, tobacco and black nightshade HypSys's all induce the production of protease inhibitors when supplied through cut petioles. Furthermore, the tomato preproHypSys gene is induced upon wounding of leaves and upon systemin or JA treatment of the leaves (Pearce et al., 2001, 2009; Pearce and Ryan, 2003). In contrast, the petunia HypSys's showed no induction of protease inhibitors against herbivore attacks, however they did induce the expression of *defensin 1* (Pearce et al., 2007). *Defensin 1* is a gene associated with innate immunity in plants, suggesting that the petunia HypSys's still appear to function in plant defense signaling pathways. The petunia HypSys's are functionally more similar to the AtPep family of peptides than to the other HypSys's implying that there may be some functional diversity within peptide families specific to some plants (Bhattacharya et al., 2013). The StHypSys's are functionally comparable to the tobacco and tomato HypSys peptide, however they appear to have additional functions as they also activate enzymes to protect against oxidative stress and free-radical generation (Bhattacharya et al., 2013). These are both outcomes of herbivore attack and pathogen infection therefore the StHypSys peptides appear to act as defense elicitors against both insects and pathogens (Bhattacharya et al., 2013). Although the experiments were not done it could be expected that pre-treatment of plants with HypSys prior to insect attack or pathogen infection would result in reduced symptoms as the defense pathways are primed.

#### Pep1 and its receptor: The model plant arabidopsis gives up its first peptide

Pep1 from *A. thaliana* is another peptide which is associated with the activation of the plant defense system (Huffaker et al., 2006). This peptide consists of 23 AA residues and is derived from the C-terminus of a 92-AA precursor protein called PROPEP1 (Huffaker et al., 2006; Farrokhi et al., 2008; Table 1). AtPROPEP1 shows low level expression in all tissue types but increased expression is evident when plants are wounded or treated with methyl jasmonate (MeJA) or ethlylene (ET) (Huffaker et al., 2006). This suggests a role for this protein in plant defense. Plants treated through cut petioles with 20 nM AtPep1, showed increased expression of *Plant Defensin 1.2* (*PDF1.2*)*, Pathogenesis-related Protein 1* (*PR-1*) and *PROPEP1* along with the production of H_2_O_2_ (Huffaker et al., 2006; Huffaker and Ryan, 2007). The production of H_2_O_2_ and *PDF1.2* are both associated with innate immunity in plants while *PR-1* is known to be induced during Pathogen-Associated Molecular Patterns (PAMP) Triggered Immunity (PTI) however its function remains unknown. Huffaker et al. (2006) and Huffaker and Ryan (2007) used mutants for the JA and ET pathways, to show that AtPep1 functions upstream of the JA/ET pathways, as expression of *PDF1.2* and *PR-1*, triggered by the peptides, was blocked. Transgenic plants constitutively overexpressing *AtPROPEP1* showed an increase in root mass compared to WT plants. When these overexpressing plants were infected with the root pathogen *Pythium irregular* the increase in root growth compared to infected WT plants was still apparent however, the aerial parts of the plants show no difference in growth or disease symptoms between the transgenic and WT. This implies that overexpression of *PROPEP1* provides a growth advantage to the roots even in the presence of a pathogen (Huffaker et al., 2006). This growth advantage looks to be a by-product of *PROPEP1* overexpression as the aerial components of the plants do not show increased growth implying that nutrient acquisition has not increased. Six annotated homologs of the precursor protein have been identified in *A. thaliana*, two of which (PROPEP2 and 3) show increased expression when plants are infected with bacterial, fungal and oomycete pathogens (Huffaker et al., 2006). The expression of *PDF1.2* and *PR-1* was increased in plants treated with AtPep1 (Huffaker et al., 2006). All of these results indicate that *PROPEP1-3* has a role in a feedback loop for defense signaling that is activated in the presence of pathogens and also increases root development (Huffaker and Ryan, 2007).

Orthologs of these *A. thaliana* genes have since been found in other plant species suggesting an important and ancient role for these peptides. Important crop species are among those to be investigated for the presence of Pep peptides. Recently an ortholog of *AtPROPEP1* was identified in *Solanum lycopersicum, SlPROPEP*, which has high AA identity (96%) with the C-terminal region of the *S. tuberosum* gene (Trivilin et al., 2014). Silencing of the tomato ortholog increased the susceptibility of plants to *Pythium dissotocum* but also decreased the expression of key defense response genes e.g., *PR-1, PR-5, ERF1, LOX-D*, and *DEF2* (Trivilin et al., 2014). These results showed that SlPROPEP plays a role in resistance against *P. dissotocum*. Huffaker et al. (2011) identified and characterized an ortholog of *AtPROPEP1* in maize. Similar to *AtPROPEP1, ZmPROPEP1* is induced in response to JA or peptide treatment, but also fungal infection. In maize plants treated with ZmPep1 it was noted that the expression of several defense genes were up-regulated, *endochitinase A, PR-4, PRms, SerPIN*, and *Benzoxazineless1* (Huffaker et al., 2011). Interestingly, this work also showed that pre-treatment of maize leaves with 25 pmol of ZmPep1 increased resistance of leaves to *Cochliobolis heterostrophus* and pre-treatment of stalks with 5 nmol of ZmPep1 increased resistance to *Colletotrichum graminicola* (Huffaker et al., 2011). The work described above clearly shows a benefit to the plants when pre-treated with these peptides in reducing pathogenicity. Therefore, these peptides may be useful for crop plants in areas that are known to have high pathogenicity instead of the use of fungicides or pesticides by either foliar application or with the generation of transgenic crop plants.

Two cell surface receptors for AtPep1, PEPR1 and PEPR2, have been identified in *A. thaliana* and demonstrated to be membrane associated leucine rich repeat (LRR) receptor kinases (Yamaguchi et al., 2006, 2010). These two receptors are 76% similar at the protein level and both showed induced expression upon wounding, also following exogenous application of Pep1 peptide and MeJA (Yamaguchi et al., 2010). PEPR1 was confirmed as recognizing Pep1 resulting in the activation of plant defense responses (Yamaguchi et al., 2006). Gain of function was also observed when *AtPEPR1* was overexpressed in transgenic tobacco cell lines resulting in the rapid alkalinization of the media upon treatment with AtPep1 (Yamaguchi et al., 2006). Like previously described experiments, pre-treatment of *A. thaliana* with exogenous Pep1 resulted in increased resistance to *Pseudomonas syringae pv. tomato DC3000* (Yamaguchi et al., 2010). PEPR1 and PEPR2 show limited redundancy as single mutants of each showed reduced resistance to *P. syringae pv. tomato* while a double mutant is susceptible to *P. syringae pv. tomato* (Yamaguchi et al., 2010). This work has elegantly identified the cell surface receptor of a secreted plant peptide which induces defense responses against pathogen invasion.

#### Soybean peptides: Soybean is potentially the next high peptide yielding plant

Whilst tomato and tobacco have traditionally been used for peptide discovery, soybean is rapidly becoming the new dicot model as a source of new peptides. Recently many new peptide families have been identified in soybean, GmPep914 and GmPep890 are two recently identified peptides shown to cause the rapid alkalinization of cell culture medium (Yamaguchi et al., 2011). These peptides consist of 8 AA residues and are derived from the C-terminal end of 52-AA precursor proteins GmPROPEP914 and GmPROPEP890 (Yamaguchi et al., 2011; Table 1). Treatment of soybean leaves with GmPep914 and GmPep890 induced the expression of their precursor proteins as well as defense genes including *CYP93A1*, a cytochrome P450 gene, *chitinaseb1-1* and *Gmachs1* (*Glycine max chalcone synthase1*) which is involved in phytoalexin production (Yamaguchi et al., 2011). GMPep914 is unrelated to any of the previously identified peptides with a known function in plant defense and it is also the smallest peptide found to have such a role (Yamaguchi et al., 2011).

GmSubPep was also recently identified in soybean. The sequence of this 12-AA peptide was found within an extracellular protein family called subtilisin-like protease (subtilase), specifically in the gene *Glyma18g48580* (Pearce et al., 2010; Table 1). This peptide appears unique for soybean as systemin is for tomato due to the GmSubPep sequence located in the protease-associated (PA) domain of *Glyma18g48580* which is a unique region of the subtilases within legumes (Pearce et al., 2010). Also when this peptide was tested on tomato, tobacco, *Arabidopsis* and corn cell cultures no alkalinization was observed (Pearce et al., 2010). The receptor of Glyma18g48580 was not induced upon wounding or treatment with MeJA, however treatment of cell cultures with the peptide resulted in an increase in some defense genes (*Cyp93A1, Chib-1b, and PDR12;* Pearce et al., 2010). Glyma18g48580 is predicted to be an apoplastic protein of unknown function and it has been speculated that this protein comes into contact with non-self molecules, due to its apoplastic localization. This in turn could induce cleavage of the peptide resulting in activation of defense signaling pathways. No work has been done so far with these soybean peptides to determine if they are able to increase plant resistance to pathogens or insects but given that defense genes are up-regulated it is conceivable that this is the case.

#### CAPE1: Unraveling the potential function of PR-1

The CAPE1 peptide, was recently isolated from tomato and has been shown to be a Damage-Associated Molecular Pattern (DAMP) elicitor (Chen et al., 2014). CAPE1 was identified using endogenous peptide mixtures extracted from stressed and unstressed plants, these extracts were analyzed by nanoflow ultrahigh performance liquid chromatography mass spectrometry (nanoUHPLC-MS). CAPE1 is an 11-AA peptide derived from the C-terminus of a 159-AA precursor protein called PR-1b (also known as P14a; Chen et al., 2014; Table 1). Expression analysis showed that CAPE1 was induced by wounding and that treatment of tomato plants with the CAPE1 peptide resulted in the induction of defense hormones such as JA and salicylic acid (SA) as well as increased expression of several pathogen-related marker genes, *PR-2, PR-7* and *PR1b* (Chen et al., 2014). However, CAPE1 treated plants did not induce PTI-responsive genes, suggesting that CAPE1 may induce systemic resistance rather than PTI (Chen et al., 2014). The authors also showed that pre-treatment of tomato leaves with CAPE1 prior to infection with *P. syringae pv. tomato* resulted in reduced disease symptoms in the absence of Hypersensitive Response (HR) (Chen et al., 2014). CAPE1 also showed an anti-herbivore activity; *Spodoptera litura* larvae feeding experiments using CAPE1 pre-treated tomato leaves resulted in a 20% reduction in size and weight (Chen et al., 2014). This data demonstrates that CAPE1 has antibacterial and anti-herbivore activity. Curiously anti-fungal activity was not reported on. Comparing these pre-treatment results to those discussed above for the ZmPep1 peptide (Huffaker et al., 2011) shows that these peptides appear to prime plants for pathogen resistance.

Sequence analysis has shown that the CAPE1 peptide is conserved in many flowering plants. The authors also suggest that three AAs immediately in front of the peptide may be the cleavage site (CNYx) and this could act as a conserved motif that could indicate a bio-active peptide in other species (Chen et al., 2014). Pathogenesis-related protein 1 from *A. thaliana* (AtPR1) contains the CAPE1 and potential bio-active motifs (CNYx. PxGNxxxxPY) but has the least protein similarity to tomato PR-1b. However, this CAPE1 peptide from AtPR1 was shown to increase immunity of *A. thaliana* to *P. syringae pv. tomato* indicating that this peptide functions similarly to the CAPE1 from tomato PR-1b (Chen et al., 2014).

The authors suggest a motif that could be used to search other RNAseq or genome database to investigate whether the motif is used in other peptide cleavage sites, showing a downstream application of this technique. As for the plant resistance increase that is observed when the plants are pre-treated with CAPE1 it gives further information about the PR1 protein which has always been associated with plant defense but a function of this protein has been elusive. Since PR1 is present in most plant species this could be a good biocontrol agent against pathogens and shows an ancient and probably conserved role for this protein.

#### Inceptins: Insect feeding aids plant defense

Inceptins fall into a small category of plant peptides as they are processed by enzymes from the invading organism, not by the plant itself. Inceptins are 11–13 AA acidic peptides which contain disulphide bridges and are derived from the chloroplastic ATP synthase γ-subunit (Schmelz et al., 2006; Table 1). These peptides were identified from the saliva of larval fall armyworms (*Spodoptera frugiperda*) that were feeding on cowpea (*Vigna unguiculata*) plants. Inceptin peptides are cleaved from the chloroplastic ATP synthase γ-subunit during feeding of the larvae on the leaves; the peptide in the saliva of *S. frugiperda* is then detected by the plant, resulting in the activation of defense related genes (Schmelz et al., 2006). The perception of inceptin by cowpea plants results in activation of the volatiles homoterpene (E)-4,8-dimethyl-1,3,7-nonatriene (DMNT) and cinnamic acid which are known to attract natural insect enemies but also results in induction of JA and SA hormones (Schmelz et al., 2006, 2007).

### Defensins: Peptides endogenous to all plant species

Plant defensins are produced in all plant species and are either constitutively expressed in storage and reproductive organs or can be induced during biotic or abiotic stress (Vriens et al., 2014). These peptides are known as cationic peptides and are usually 45–54 AAs in length. These peptides typically harbor a cysteine-stabilized αβ-motif (CSαβ) which has an α-helix and a triple-stranded antiparallel β-sheet which is stabilized by four disulphide bridges (Vriens et al., 2014).

There are typically two groups of plant defensins based on the proproteins. Both groups contain a signal peptide which targets the protein to the endoplasmic reticulum where the mature protein is folded and enters the secretory pathway. One of these groups contains an additional prodomain on the C-terminus which is cleaved during processing through the secretory pathway (Vriens et al., 2014). Some well-known plant defensins are RsAFP1 and RsAFP2 from the seeds of radish (Terras et al., 1992; Table 1), MsDef1 and MtDef4 from the seeds of *Medicago* species (Gao et al., 2000; Ramamoorthy et al., 2007; Sagaram et al., 2013), NaD1 from the flowers of tobacco (Lay et al., 2003a,b) and Psd1 from seeds of pea pods (Almeida et al., 2000). Each of these peptides has antifungal activity but so far very few defensins have been found with antibacterial activity. In recent years the use of transgenics has been employed to investigate some defensins for their ability to protect plants from fungal infection or insect wounding. Transgenic rice plants containing a plant defensin from *Brassica rapa*, BrD1, showed increased resistance to the insect brown plant hopper (Choi et al., 2009). Transgenic tomato plants containing MsDef1 showed increased seedling resistance to *F. oxysporum* f. sp. *Lycopersici* (Abdallah et al., 2010). While transgenic tobacco and potato plants containing the defensin NmDef01 from *N. megalosiphon* showed increased resistance to the oomycete *Phytophthora infestans* in both glasshouse and field trials (Portieles et al., 2010). The mode-of-action and pathogenicity experiments of two defensins, RsAFPs and NaD1, will be discussed here in more detail.

*Raphunus sativus* antifungal protein 1 (RsAFP1) and RsAFP2 were first isolated from radish seeds by use of ammonium sulfate fractionation and anion-exchange chromatography and were found to produce 5 kDa peptides which are assembled and stabilized by disulfide bridges (Terras et al., 1992). The RsAFP1 peptide is 44 AAs in length and RsAFP2 is 36 AAs in length (Terras et al., 1992). Within the first 36 AAs of these peptides there is a high amount of conservation with only 2 AAs difference. These changes though are significant and result in a higher positive charge for RsAFP2 compared to RsAFP1. RsAFP2 has a low half maximal inhibitory concentration (IC_50_) range of 0.4–25 pg/ml when tested against plant pathogenic fungi while RsAFP1 has a larger IC_50_ range of 0.3–100 pg/ml. The antifungal activity of RsAFP2 is also more efficacious compared to RsAFP1 in the presence of salts (Terras et al., 1992). RsAFP2 interacts with fungal glucosylceramide (GlcCer) found in the membrane and cell wall of fungal cells (Thevissen et al., 2004). GlcCer is usually associated with other components which form lipid rafts in fungal membranes and cell walls (Vriens et al., 2014). The binding to GlcCer results in activation of MAP kinase and cell wall integrity signaling pathways, the production of Reactive Oxygen Species (ROS), induction of ion fluxes and activation of caspases, resulting in abnormal hyphal growth and division (De Samblanx et al., 1997; Navarro-García et al., 2005; Aerts et al., 2009; Vriens et al., 2014). The ability of RsAFP2 to inhibit fungal infection of whole plants was tested with the generation of stable transgenic wheat constitutively expressing RsAFP2 (Li et al., 2011). Compared to untransformed control plants, the transgenic RsAFP2 plants showed increased resistance to *Fusarium graminearum* and *Rhizoctonia cerealis* in glasshouse experiments over several generations and in field trials for T_4_ and T_5_ generations (Li et al., 2011). The authors also noted that this resistance was heritable (Li et al., 2011). Another well investigated defensin is *Nicotiana alata* Defensin 1 (NaD1; Table 1). NaD1 is one of the rarer plant defensins as it was isolated from the flower of ornamental tobacco (*Nicotiana alata*) and not the seed, where most defensins have been found. NaD1 is only active against filamentous fungi and has no effect against bacteria, yeast or human cell lines (van der Weerden et al., 2008). The fungal membrane ligand for NaD1 has recently been identified as phospholipid phosphatidylinositol 4,5-bisphosphate (PIP2; Poon et al., 2014). These authors demonstrated that NaD1 oligomers interact with the head group of two PIP2 molecules and that this complex is required for membrane permeabilization and subsequent cell death (Poon et al., 2014). If the NaD1 oligomer structure is disrupted then antifungal activity is also lost (van der Weerden et al., 2008; Poon et al., 2014). Once the fungal cell wall has been disrupted NaD1 has been shown to form an aperture of 14–23 Å in size through which it may enter the cytoplasm (van der Weerden et al., 2008). It has been shown that NaD1 is able to localize to the cytoplasm of hyphal cells and these cells subsequently show granulation of the hyphal cytoplasm and cell death (van der Weerden et al., 2008). This indicates that defensins may not just target the cell membrane, but also have intracellular functions. Once NaD1 is inside the cytoplasm of hyphal cells, ROS production occurred within these cells suggesting that cell death is occurring.

There is still more to be learnt about the mode-of-action of NaD1 but this defensin has great potential as a natural anti-fungal in plants other than tobacco. This has been elegantly demonstrated by Gaspar et al. (2014), where they generated transgenic homozygous cotton lines constitutively overexpressing NaD1. These lines were tested in greenhouse assays for resistance to *F. oxysporum* f. sp. *vasinfectum* (*Fov*) with one line being chosen for field trials in soil naturally infected with *Fov* and *Verticillium dahlia* (Gaspar et al., 2014). These greenhouse assays and field trials of NaD1 expressing transgenic plants showed an increase in resistance and cotton production compared to the non-transgenic parental line when *Fov* and *V. dahlia* are present in the soil (Gaspar et al., 2014). The NaD1-expressing plants also showed no detrimental agronomic properties compared to the control non-transgenic parental lines. This is a good example of a defensin taken from one plant species and used as a biocontrol agent in an economically important crops species against soil borne fungi.

All of the peptides discussed above have shown a direct correlation with the induction of the plant defense pathways to protect against herbivores and/or pathogens. In some cases experimental data is available to show a decrease in infection of plants when pre-treated with these peptides. This is a small number of plant defense peptides and without doubt there are many more to be identified. As yet undiscovered peptides may be species specific while others could be ancient defense signals active in all higher plants.

## AMPs secreted by non-plant organisms, and their potential for plant pathogen resistance engineering

Whilst plants have proven (and will continue to do so) a rich source of antimicrobial peptides, non-plant organisms are also subject to pathogen attack and synthesize AMPs as a defense mechanism. These non-plant AMPs can in turn be either sprayed on crops or artificially introduced into crop species to engineer pathogen resistance using transgenic methods. Tables 2 and 3 summarizes the non-plant AMPs discussed in this review, detailing their class and molecular weight, the analytical methods employed for their identification in the publications cited here, the organisms naturally secreting them, the targeted pathogens and plant hosts.

**Table 2:** **Non-plant nonribosomal AMPs**.

**Peptide name per taxa of origin**	**AA length**	**Mr**	**Peptide analysis**	**Origin species**	**Target pathogens**	**Plant host**	**References**
**BACTERIUM GRAM −**							
2-amino-3-(oxirane-2,3-dicarboxamido)-propanoyl-valine (APV)	3	317	LC-ESI-MS/MS, NMR	*Pantoea agglomerans* 48b/90 (Pa48b)	*Agrobacterium tumefaciens, Erwinia amylovora, Pseudomonas syringae pathovars, Serratia marcescens, Bacillus subtilis*	None tested	Sammer et al., 2009
2-amino-3-(oxirane-2,3-dicarboxamido)-propanoyl-valine (APV)	3	317	Not in this study	*Pantoea agglomerans* 48b/90 (Pa48b)	*Pseudomonas syringae* pv. *glycinea*	Soybean	Sammer et al., 2012
syringolin		493	FAB-MS, ESI-MS, NMR	*Pseudomonas syringae* pv. *syringa*	*Pyricularia oryzae*	Rice	Wäspi et al., 1998
Syringolin A			Not in this study	*Pseudomonas syringae* pv. *syringa*	*Blumeria graminis* f sp. *tritici*	Wheat	Wäspi et al., 2001
Syringomycin E (SRE)		1225	HPLC-ESI-Q-MS	*Pseudomonas syringae* pv. *syringae* strain B359	*Fusarium oxysporum, Verticillium dahliae, Botrytis cinerea, Penicillium expansum, Phytophthora infestans, and Rhodotorula pilimanae*	Apple	Fogliano et al., 2002
Syringopeptin 25A (SP25A)		2401	HPLC-ESI-Q-MS	*Pseudomonas syringae* pv. *syringae* strain B359	*Fusarium oxysporum, Verticillium dahliae, Botrytis cinerea, Penicillium expansum, Phytophthora infestans, and Rhodotorula pilimanae*	Apple	Fogliano et al., 2002
**BACTERIUM GRAM +**							
Bacillomycins D		1073, 1059, 1045	HPLC-ESI-MS/MS	*Bacillus amyloliquefaciens* Q-426	*Fusarium oxysporum* f. sp. *spinaciae*	None tested	Zhao et al., 2014
Fengycins A		1464, 1450	HPLC-ESI-MS/MS	*Bacillus amyloliquefaciens* Q-426	*Fusarium oxysporum* f. sp. *spinaciae*	None tested	Zhao et al., 2014
Fengycins B		1506, 1478	HPLC-ESI-MS/MS	*Bacillus amyloliquefaciens* Q-426	*Fusarium oxysporum* f. sp. *spinaciae*	None tested	Zhao et al., 2014
7-O-malonyl macrolactin A		488	LC-ESI-Q-Trap-Ms/MS	*Bacillus amyloliquefaciens* strain NJN-6	*Fusarium oxysporum* f. sp. *cubense, Ralstonia solanacearum*	Banana	Yuan et al., 2012
7-O-succinyl macrolactin A		502	LC-ESI-Q-Trap-Ms/MS	*Bacillus amyloliquefaciens* strain NJN-6	*Fusarium oxysporum* f. sp. *cubense, Ralstonia solanacearum*	Banana	Yuan et al., 2012
Bacillomycins D	7	1031, 1045	LC-ESI-Q-Trap-Ms/MS	*Bacillus amyloliquefaciens* strain NJN-6	*Fusarium oxysporum* f. sp. *cubense, Ralstonia solanacearum*	Banana	Yuan et al., 2012
Fengycin			Not in this study	*Bacillus amyloliquefaciens* strain PPCB004	*Alternaria citri, Botryosphaeria* sp., *Colletotrichum gloeosporioides, Fusicoccum aromaticum, Lasiodiplodia theobromae, Penicillium crustosum, and Phomopsis perse*	Orange	Arrebola et al., 2010
Iturin A			Not in this study	*Bacillus amyloliquefaciens* strain PPCB004	*Alternaria citri, Botryosphaeria* sp., *Colletotrichum gloeosporioides, Fusicoccum aromaticum, Lasiodiplodia theobromae, Penicillium crustosum, and Phomopsis perse*	Orange	Arrebola et al., 2010
Surfactin			Not in this study	*Bacillus amyloliquefaciens* strain PPCB004	*Alternaria citri, Botryosphaeria* sp., *Colletotrichum gloeosporioides, Fusicoccum aromaticum, Lasiodiplodia theobromae, Penicillium crustosum, and Phomopsis perse*	Orange	Arrebola et al., 2010
Fengycins A (C15–C17)		1464, 1477	MALDI-TOF MS	*Bacillus atrophaeus* CAB-1	*Botrytis cinerea, Sphaerotheca fuliginea*	Cucumber	Zhang et al., 2013
Mycosubtilin			Not in this study	*Bacillus subtilis*	*Bremia lactucae*	Lettuce	Deravel et al., 2014
Surfactin			Not in this study	*Bacillus subtilis*	*Bremia lactucae*	Lettuce	Deravel et al., 2014
Fengycins		1435, 1505	HPLC-ESI-MS	*Bacillus subtilis* strain M4	*Pythium ultimum, Botrytis cinerea*	Bean, apple	Ongena et al., 2005
Iturin			HPLC-ESI-MS	*Bacillus subtilis* strain M4	*Pythium ultimum, Botrytis cinerea*	Bean, apple	Ongena et al., 2005
Surfactin			HPLC-ESI-MS	*Bacillus subtilis* strain M4	*Pythium ultimum, Botrytis cinerea*	Bean, apple	Ongena et al., 2005
Fusaricidins A, B, and C		884, 898, 948	MALDI FT-ICR MS imaging	*Paenibacillus polymyxa* strain Pp56	*Rhodotorula aurantica, Botrytis cinerea, Fusarium oxysporum, Cladosporium cucumerinum, Phythium aphanidermatum, Pseudomonas syringae*	None tested	Debois et al., 2013
LI-F lipopeptides		912, 926	MALDI FT-ICR MS imaging	*Paenibacillus polymyxa* strain Pp56	*Rhodotorula aurantica, Botrytis cinerea, Fusarium oxysporum, Cladosporium cucumerinum, Phythium aphanidermatum, Pseudomonas syringae*	None tested	Debois et al., 2013
**FUNGI**							
Trichokonin VI (TK VI)			Not in this study	*Trichoderma pseudokoningii* strain SMF2	*Fusarium oxysporum*	None tested	Shi et al., 2012
Trichokonin (TK)			Not in this study	*Trichoderma pseudokoningii* strain SMF2	*Pectobacterium carotovorum* subsp. *carotovorum*	Chinese cabbage	Li et al., 2014

**Table 3:** **Animal ribosomal AMPs**.

**Peptide name**	**AA length**	**Mr**	**Peptide analysis**	**Origin Species**	**Target pathogens**	**Plant host**	**References**
Penaeidin4-1 (Pen4-1)	47		Not in this study	*Litopenaeus setiferus* (shrimp)	*Sclerotinia homoecarpa, Rhizoctonia solani*	*Agrostis stolonifera*	Zhou et al., 2011
Metchnikowin (Mtk isoforms A and B)	26	3025, 3045	HPLC-ESI-MS, Edman	*Drosophila melanogaster*	*Micrococcus luteus, Neurospora crassa*	None tested	Levashina et al., 1995
Metchnikowin (Mtk isoforms A and B)	26		Not in this study	*Drosophila melanogaster*	*Fusarium graminearum*	Barley	Rahnamaeian et al., 2009
Metchnikowin (Mtk isoforms A and B)	26		Not in this study	*Drosophila melanogaster*	*Blumeria graminis* f. sp. *hordei*	Barley	Rahnamaeian and Vilcinskas, 2012
Thanatin	21	2436	2-D HPLC (CXC-RP)-ESI-MS	*Podisus maculiventris* (stinkbug)	*Magnaporthe oryzae*	Rice	Imamura et al., 2010
Thanatin			Immunoblots	*Podisus maculiventris* (stinkbug)	*Aspergillus flavus*	Maize	Schubert et al., 2015
Cecropin A	37	4000	Immunoblots, SDS-PAGE shotgun	*Hyalophora cecropia* (moth)	*Fusarium verticillioides, Dickeya dadantii*	Rice	Bundó et al., 2014
Magainin-2 (mag)	23		Not in this study	*Xenopus laevis* (frog)	*Sclerospora graminicola*	Pearl millet	Ramadevi et al., 2014
Cathelicidin (LL-37 Met37Leu)	37	4000	Immunoblots	*Homo sapiens* (Human)	*Pectobacterium carotovorum* subsp. *carotovorum, Fusarium oxysporum* f. sp. *Lycopersici, Colletotrichum higginsianum, Rhizoctonia solani*	Chinese cabbage	Jung et al., 2012
Esculentin-1 (Esc28L Met-28Leu)	46	5513 (with SP)	Immunoblots, LC-MALDI-TOF	*Rana esculenta* (frog)	*Pseudomonas syringae* pv. *Tabaci, Pseudomonas aeruginosa, Phytophthora nicotianae*	Tobacco	Ponti et al., 2003

### Nonribosomal AMPs identified from microbes and tested by exogenous application

Biological control of plant pests through the use of natural antagonistic microorganisms producing a vast array of AMPs has emerged as a promising alternative to reduce the use of chemical pesticides. Cyclic lipopeptides are nonribosomal AMPs predominately produced by *Bacillus* and *Paenibacillus* spp. and increasing numbers of lipopeptides are being isolated from *Pseudomonas* spp. as well (Patel et al., 2015). The following section describes the most favorable AMPs for crop disease resistance in such bacteria as well as in fungi.

#### Syringolin, syringomycin, and syringopeptin from *Pseudomonas* sp. bacteria

Syringolin A is a peptide secreted by the Gram-negative bacterium *P. syringae* pv. *syringae*. Syringolin A has a ring structure composed of 5-methyl-4-amino-2-hexenoic acid and 3,4-dehydrolysine (Wäspi et al., 1998; Table 2). The α-amino group is joined by a peptide bond to a valine linked to another valine via a urea moiety (Wäspi et al., 1998). Syringolin A was recovered from liquid cultures by centrifugation, filtering, ultra-filtration, followed by gel filtration chromatography. The application of HPLC-purified syringolin onto *Oryza sativa* (rice) detached leaves induced resistance toward the fungal plant pathogen *Pyricularia oryza* (rice blast disease). Plate assays showed that syringolin A did not directly affect the growth of *P. oryzae*. Furthermore, no visible phytotoxic effects were observed when applied on detached rice leaves at the highest concentration (0.05 mM). Gene expression analysis of detached leaves sprayed with syringolin A solution and subsequently inoculated with *P. oryzae* indicated the increased transcript abundance of defense genes (including *Pir7b, Pir2, Pir2*, and *Rir1* mRNAs). Consequently, it was proposed that syringolin A elicits rice defense responses through acquired resistance rather than being directly anti-fungal (Wäspi et al., 1998). Subsequently, syringolin A was trialed in other pathosystems. *Triticum aestivum* (wheat) detached leaves were sprayed with a syringolin A solution 2 days prior to inoculation with *Blumeria graminis* f. sp *tritici* (powdery mildew; Wäspi et al., 2001). As previously observed with rice blast disease, wheat leaves exposed to syringolin A prior to fungal infection appeared more resistant. Curative effects of syringolin A were also reported in this study with whole wheat plants first infected with powdery mildew and then sprayed with a syringolin A solution. Fungal colonization of wheat tissues was arrested if syringolin A was exogenously applied 2 days post-inoculation. This study suggests that the mode-of-action of syringolin A either targets the host cells in a way that maintains host's hypersensitivity or reverses the suppression of host defense imposed by the pathogen (Wäspi et al., 2001).

Cyclic lipodepsipeptides (LDPs) include two other AMPs secreted by *P. syringae* pv. *syringae*, syringomycin E (SRE) and syringopeptin 25A (SP25A) (Table 2). SRE is formed by nona-peptide lactones acylated with a long-chain 3-hydroxy FA. SP25A is composed of long and highly hydrophobic peptide chain, with a polar lactonized penta- or octa-peptide moiety at the C terminus. It has been speculated that pathogen cell walls would present a natural barrier which impairs peptide anti-microbial activity, therefore making pest cell wall porous would enhance AMPs action (Fogliano et al., 2002). LDPs were extracted from centrifuged liquid cultures followed by acetone precipitation and further fractionated using reverse-phase (RP) HPLC. SRE and SP25A were identified by MS. SRE and SP25A, together with cell wall degrading enzymes (CWDEs) from *Trichoderma atroviride*, were incorporated into *in vitro* assays performed on liquid cultures of *F. oxysporum, V. dahliae, Botrytis cinerea, Penicillium expansum, Phytophthora infestans*, and *Rhodotorula pilimanae* (Fogliano et al., 2002; Table 2). These analyses revealed that while SRE or SP25A alone did not inhibit fungal growth, in the presence of endochitinase and/or glucanase SRE and SP25A prevented spore germination, thus unraveling synergism between LDPs and CWDEs. Furthermore, postharvest assays performed on wounded apple fruits first inoculated with *B. cinerea*, then with *P. syringae*, and/or with *T. atroviride* indicated that the least number of *B. cinerea* related-wounds were observed when both *P. syringae* and *T. atroviride* were combined (Fogliano et al., 2002). This study thus provided support for a synergistic interaction between *P. syringae* LDPs and *T. atroviride* CWDEs against a variety of fungi, thereby supporting the hypothesis that, in a biocontrol strategy, the efficacy of AMPs in plant pathogen resistance would be improved if pathogen cell walls were also made vulnerable (Fogliano et al., 2002). This experiment however has not validated which LDPs and CWDEs are responsible for the synergism observed *in vivo*.

#### Fengycins, iturins, surfactins, bacillomycins, macrolactin, and mycosubtilin from *Bacillus* sp. bacteria

Potent antifungal lipopeptides such as surfactins, iturins and fengycins are produced by some strains of the Gram-positive bacterium *Bacillus subtilis* such as strain M4 which are beneficial rhizobacteria (Ongena et al., 2005; Table 2). *B. subtilis* strain M4 was also found to secrete various fengycin homologs, with the great majority of them harboring a C16 or C17 FA chain (Ongena et al., 2005). An enriched lipopeptide extract was obtained by Solid Phase Extraction (SPE) of the crude cell-free culture broth of M4 strain eluted using methanol, followed by HPLC separations and ElectroSpray Ionization (ESI)-MS analyses. *In vitro* assays showed that the lipopeptide-enriched supernatant had a strong inhibitory effect on the growth of *F. oxysporum, Pythium ultimum, Rhizoctonia solani, Rhizopus sp*., and *B. cinerea*. The antimicrobial potential of these peptides has also been described *in planta*. Post-harvest assays on immature apple fruits preconditioned with M4 supernatant or the lipopeptide-enriched supernatant showed increased pathogen resistance, mostly attributable to fengycins (Ongena et al., 2005). Similarly, bean seedlings with roots pre-treated with M4 supernatant prior to *B. cinerea* leaf inoculation displayed reduced disease symptoms. *B. subtilis* M4 thus shows great potential as biocontrol agent to better manage soilborne, foliar and post-harvest diseases. The lipopeptide modes-of-action, in particular that of fengycins, rely not only on the direct inhibition of the plant pathogen on infected plant organs, but also on an indirect interaction mediated through the host plant via systemic resistance induction. Direct interaction was demonstrated through the disease control provided by treatment of fruits with lipopeptide-enriched supernatant and by *in situ* detection of fengycins in inhibitory amounts. Indirect interaction was mediated by the production of phenolic compounds involved in or derived from the defense-related phenylpropanoid metabolism upon pathogen attack (Ongena et al., 2005).

More recently the same set of secreted lipopeptides, fengycins, iturins, and surfactins, along with bacillomycin, was detected in the Gram-positive bacterium *Bacillus amyloliquefaciens* strain PPCB004. Bacteria were cultured in liquid medium; lipopeptides were extracted using n-butanol, followed by complete evaporation and resuspension in methanol. Growth assays showed that these peptides had inhibitory properties on the mycelial growth and/or spore germination of various post-harvest fungal pathogens, including *Alternaria citri, Botryosphaeria sp., Colletotrichum gloeosporioides, Fusicoccum aromaticum, Lasiodiplodia theobromae, Penicillium crustosum*, and *Phomopsis perse* (Arrebola et al., 2010; Table 2). In this study, iturin A demonstrated the strongest inhibitory effect. Post-harvest assays performed on orange fruits inoculated with *A. citri* and *C. gloeosporioides* displayed less disease incidence when treated with *B. amyloliquefaciens* strain PPCB004 either before or after inoculation. The mode-of-action for iturin A has been proposed to disrupt the fungal cytoplasmic membrane, creating transmembrane channels, resulting in the release of vital ions such as K^+^, thus preventing spore germination and impairing mycelium development (Arrebola et al., 2010).

The lipopeptides bacillomycin D, fengycins A, and B are secreted by *B. amyloliquefaciens* strain Q-426 (Zhao et al., 2014; Table 2). They were recovered from cell-free supernatant of liquid cultures, further filtered and acid precipitated, prior to drying and methanol resuspension. Using two-dimensional (2-D) HPLC separation and tandem MS (MS/MS) analyses their molecular structure was elucidated. The peptide moiety comprised of 7–10 AA residues arranged in a cyclic structure, while the lipid moiety was composed of a chain of 14–17 FAs. Fengycin A purified from strain Q-426 disrupted the germination of spores from the fungal pathogen *F. oxysporum* f. sp. *spinaciae* in a dose dependent manner, with complete inhibition above 50 ug/mL. Fengycin A also modified hyphal growth, albeit without affecting cell membrane permeability (Zhao et al., 2014).

Along with iturin A, isoforms of bacillomycin D and of the macrolactin family, macrolactin A, 7-O-malonyl macrolactin A, and 7-O-succinyl macrolactin A were identified by HPLC-MS/MS analyses from the supernatant of centrifuged liquid cultures of *B. amyloliquefaciens* strain NJN-6 (Yuan et al., 2011, 2012; Table 2). This strain was isolated from the root system of a healthy banana plant. Plate assays showed that both purified bacillomycin D isoforms inhibited the hyphal growth of *F. oxysporum* f. sp. *cubense*, a banana fungal pathogen. Similarly, all three macrolactins inhibited the growth of Gram-negative pathogenic bacteria *Ralstonia solanacearum*. The authors demonstrate that the macrolactin activities are maintained after 1 month at room temperature (Yuan et al., 2012); such shelf-life information is valuable for an in-field application strategy. Performing tests on plant tissues infected with these pathogens would have further validated the antifungal activity of these AMPs.

Fengycin A was also secreted by the Gram-positive bacterium *Bacillus atrophaeus* strain CAB-1 and showed antagonistic effect against airborne plant fungal pathogens *B. cinerea* and *Sphaerotheca fuliginea*. Two groups of lipopeptides were identified in strain CAB-1 by MS analyses: fengycins and unknown lipopeptides. CAB-1's lipopeptide extracts obtained by centrifugation of the culture broth followed by acid precipitation yielded no detectable iturin or surfactin compounds (Zhang et al., 2013). The fengycins produced by strain CAB-1 were a mixture of isoforms with various acyl side chain lengths from C15 to C17 (Table 2). The C16 isoform of fengycin A was secreted in greater abundance than C15 and C17 isoforms and therefore would contribute more to growth inhibition of cucumber powdery mildew, *S. fuliginea*, and tomato gray mold, *B. cinerea* (Zhang et al., 2013).

Mycosubtilin is a nonribosomal cyclic lipopeptide. Surfactin and mycosubtilin were purified from *B. subtilis* strains BBG131 and BBG125, respectively. Surfactin was produced through an integrated process in a bubbleless membrane bioreactor while mycosubtilin was produced using an overflowing fed-batch process. Subsequent steps of ultrafiltration, diafiltration, evaporation and freeze-dried were required for purification (Deravel et al., 2014). Surfactin and mycosubtilin combined were reported to have dose-dependent synergetic antibiotic effects on lettuce leaves sprayed with the peptide solutions prior to inoculation with its fungal obligate pathogen *Bremia lactucae* (Deravel et al., 2014; Table 2). This data shows that combining several AMPs opens even greater possibilities in terms of biocontrol strategies, yet it also poses challenges as to designing experiments assessing various AMP combinations and engineering plants resistant to microbial pathogens.

#### Fusaricidins and LI-F lipopeptides from *Paenibacillus polymyxa* bacterium

Fusaricidins and closely related LI-F lipopeptides are synthesized by *Paenibacillus polymyxa* and display inhibitory activities against plant pathogens (Table 2). These nonribosomal peptides consist of a guanidinylated β-hydroxy FA linked to a cyclic hexapeptide including four D configured-AA residues (Debois et al., 2013). Growth inhibition effects were also observed against a variety of microbes: *Rhodotorula aurantica* (yeast), *F. oxysporum, B. cinerea, Cladosporium cucumerinum* (fungi), *Phythium aphanidermatum* (oomycetes), and *P. syringae* (Gram negative bacterium). These AMPs were identified using a matrix-assisted laser desorption ionization (MALDI) imaging approach. By using a clever experimental design involving the insertion of a sterilized MALDI glass slide coated with indium tin oxide at the bottom of the Petri dish covered with a gelified sterile nutrient medium (Debois et al., 2013). *P. polymyxa* was streaked over the glass slide and *F. oxysporum* was inoculated nearby the slide and incubated for 11 days. The glass slide was removed from the Petri dish and completely dried under vacuum prior to MALDI imaging analysis. The authors were able to obtain spectra and fragmentation patterns of the compounds released by strain Pp56 and responsible for the inhibition of *F. oxysporum* mycelial development (Debois et al., 2013). The antagonistic interaction between fusaricidins and LI-F lipopeptides and *F. oxysporum* could thus be visualized by acquiring MS spectra of the different antibiotic compounds exhibiting distinct localizations along the slide. A time-course analysis revealed the early secretion of fusaricidin B, a mixture of LI-F05b/06b/08a, and LI-F08b, away from *F. oxysporum* hyphae, thus suggesting that their production was not triggered by the presence of the fungus. These antibiotics would be readily secreted and would accumulate in toxic amount outside *P. polymyxa* cells to deter any potential pathogen attack. Indeed their distribution patterns visualized by MALDI imaging coincided with the *F. oxysporum* hyphal inhibition zone observed on the culture plates (Debois et al., 2013).

#### 2-amino-3-(oxirane-2,3-dicarboxamido)-propanoyl-valine (APV) from *Pantoea agglomerans* bacterium

APV was HPLC purified from a polar extract from liquid broth supernatant, analyzed using ESI-MS/MS and NMR, and identified as the main antibiotic compound of *Pantoea agglomerans* strain 48b/90 (Pa48b; Sammer et al., 2009). Pa48b is a Gram-negative bacterium that was isolated from soybean leaves which showed limited fire blight disease symptoms caused by the bacterium *Erwinia amylovora*. Using plate assays, APV inhibitory effect was tested against various microbial phytopathogen species, including *Agrobacterium tumefaciens, E. amylovora*, several *P. syringae* pathovars, *Serratia marcescens*, and *B. subtilis* (Table 2). APV successfully inhibited the growth of pathogens on the minimum synthetic medium in a dose dependent manner, but not on the complex medium, likely due to the presence in the latter of *N*-acetylglucosamine which would compensate for APV inhibitory effects (Sammer et al., 2009). This result suggests that the antagonistic effect of APV and *N*-acetylglucosamine on phytopathogen colonization depends on nutrient availability. In a follow-up study, the *APV* biosynthesis gene cluster was analyzed, and located onto a megaplasmid in Pa48b (Sammer et al., 2012). In this cluster, two genes are likely to be involved in APV biosynthesis regulation, whose transcription is tightly coordinated with translation to avoid precursor cytotoxicity. *In silico* sequence analysis of the *APV* gene cluster revealed a 99% identity to the diaminopropionate-peptide biosynthesis cluster of *P. agglomerans* strain CU0119. One of the genes of this cluster, *DdaI*, is predicted to be a transmembrane efflux pump mediating self-resistance to antibiotics (Sammer et al., 2012). *In planta* assays were then undertaken in which soybean leaves were first inoculated with *P. syringae* pv. *glycinea* prior to applying an APV solution onto the infected wounds. Consistent with the previous study, APV inhibitory effect was dose-dependent, yet only led to minor decrease of the disease symptoms. Therefore, APV was not confirmed to be the key antibiotic factor in the antagonism (Sammer et al., 2012).

#### Trichokonins (TK) from *Trichoderma pseudokoningii* fungus

The trichokonin (TK) family is composed of three major peptaibols, TKs VI, VII and VIII, produced by the ascomycota fungus *Trichoderma pseudokoningii* strain SMF2. Peptaibols are characterized by the presence of an unusual AA, a-aminoisobutyric acid, a C-terminal-hydroxylated and a N-terminal-acetylated AA. Because of their linear and amphipathic nature, peptaibols can form voltage-dependent ion channels in lipid bilayer membranes. TKs were obtained from solid state fermentation of *T. pseudokoningii* strain SMF2, followed by gel filtration of the crude extract and preparative HPLC separation. *In vitro* plate assays showed that TK VI exhibited antimicrobial activities against various pathogenic fungi and oomycetes, *Ascochyta citrullina, B. cinerea, F. oxysporum, Phytophthora parasitica*, and *V. dahlia* (Shi et al., 2012, Table 2). Toxicity assays using *F. oxysporum* protoplasts treated with TK VI showed the appearance of ROS, fragmentation of nuclear DNA, along with a change of fungal membrane permeability and disintegration of subcellular structures. The antimicrobial efficacy of TK was also tested on Chinese cabbage (*Brassica rapa*) leaves inoculated with the bacterial pathogen, *Pectobacterium carotovorum* subsp. *carotovorum* (Li et al., 2014). As observed by Shi et al. (2012), TK treatment led to an increase in the production of ROS, along with increased activities of pathogenesis-related proteins, and the activation of SA signaling pathway in the cabbage host. These data show that the TK family can induce cell death of pathogenic fungi and oomycetes as well as induce the activation of plant defense pathways.

### Animal ribosomal AMPs validated in transgenic plants

Unlike nonribosomal AMPs, ribosomal AMPs offer the great advantage to be synthetised by genes that can be manipulated and inserted into a plant of interest. Only a few ribosomal AMPs underwent functional validation *in planta* for pest resistance and they all originate from animal species; they are illustrated in this section.

#### Penaeidin 4-1 from shrimp

The antimicrobial peptide Penaeidin 4-1 (Pen4-1) was isolated from Atlantic white shrimp (*Litopenaeus setiferus*) under pathogen challenge. Pen4-1 is composed of 47 AAs including six cysteine residues forming three disulfide bridges (Cuthbertson et al., 2004; Table 1). Pen4-1 was purified using affinity chromatography as follows: pooled *L. setiferus* haemocyte extracts were concentrated using a SPE and applied to an affinity resin containing a Pen4-1-specific antibody. Pen4-1 can inhibit multiple plant pathogenic fungal species, such as *B. cinerea, Penicillium crustosum*, and *F. oxysporum*. Penaeidins harbor a unique two-domain structure, a proline rich N-terminal domain (PRD) and a cysteine-rich domain (CRD) with a stable alpha-helical structure, which might have contributed to its broad range of microbial targets, primarily Gram-positive bacteria and fungi. Transgenic lines of a commercial creeping bentgrass (*Agrostis stolonifera*) displayed enhanced resistance to the fungal pathogens *Sclerotinia homoecarpa* and *R. solani* as a result of Pen4-1 ectopic expression (Zhou et al., 2011; Table 3). Testing Pen4-1 into other plant hosts of *S. homoecarpa* and *R. solani* would confirm its potential as a biocontrol agent.

#### Metchnikowin (mtk), thanatin, and cecropin a from insects

Metchnikowin (mtk) is an immune-inducible peptide synthesized in the fat body of *Drosophila melanogaster* as a 52-AA pre-pro-peptide upon microbial challenges (Levashina et al., 1995; Table 3). *In vitro* assays demonstrated that mtk inhibits the growth of drosophila pathogens, the Gram-positive bacterium *Micrococcus luteus* and the ascomycete fungus *Neurospora crassa*. The mtk AA sequence was determined by use of the Edman sequencing method as a proline-rich 26-AA peptide. MS analyses revealed two mtk isoforms of 3025 and 3045 Da, respectively, due to AA substitution (Rahnamaeian et al., 2009; Table 3). Low concentrations of mtk inhibited the *in vitro* growth of the pathogenic fungi *Fusarium graminearum* and *Fusarium culmorum*. Transgenic barley plants expressing the *D. melanogaster mtk* gene in its 52-AA pre-propeptide form under the control of the inducible *mannopine synthase* (*mas*) gene promoter were produced. Mas promoter's induction was triggered by wounding, plant growth hormones, as well as fungal infection. Mtk was successfully processed into its mature form and targeted to the apoplastic compartment in transgenic plants. When inoculated with *F. graminearum*, transgenic plants displayed higher frequencies of typical defense responses such as HR of attacked cells and the development of callose deposition at the cell wall underneath attempted penetration sites. This enhanced plant innate immune response was substantiated by the up-regulation of Pathogenesis-Related genes *PR-1* and *PR-5* (Rahnamaeian et al., 2009). The activation of systemic acquired resistance (SAR) was confirmed at the transcript level in a more recent follow-up study in which the mtk barley transgenic plants were infected with *B. graminis* f. sp. *hordei* and subjected to RT-PCR analyses (Rahnamaeian and Vilcinskas, 2012; Table 3). When the transgenic barley plants were infected with *F. graminearum*, mtk treatment impeded the development of functional haustorium, whose formation is crucial for commencement of biotrophic interaction.

Thanatin is produced by the stinkbug *Podisus maculiventri*; it consists of 21 AA residues (2.4 kDa) which form an internal disulphide bond important for its antimicrobial activity (Imamura et al., 2010; Table 3). A recombinant *thanatin* gene under the constitutive control of *cauliflower mosaic virus 35S* (*CaMV35S*) gene promoter was introduced into rice plants which were then exposed to blast disease (*Magnaporthe oryzae*). Thanatin was extracted from crude extracts of the transformants, purified using 2-D HPLC (cation exchange chromatography followed by RP chromatography) and its identity confirmed by MS. Although blast disease symptoms were observed on both the transgenic lines and the WT plants, diseased areas on the transformants were significantly smaller than those on the WT plants. While transgenic rice plants were not fully protected against *M. oryzae*, they had acquired partial resistance (Imamura et al., 2010). Very recently, *thanatin* gene was introduced into maize and controlled by the *ubiquitin-1* promoter which targets the expressed AMP to the plant apoplastic space (Schubert et al., 2015; Table 3). Transgenic maize plants were grown until ears were fully developed and the mature ears were then exposed to the pathogenic fungus *Aspergillus flavus*. The expression of *thanatin* in maize transformants led to a significant reduction in fungal biomass of *A. flavus* relative to that of WT plants (Schubert et al., 2015). The mode-of-action of thanatin in plants has yet to be fully understood.

Cecropin AMPs were first isolated from the haemolymph of the moth *Hyalophora cecropia*; cecropin A (37 AAs, 4 kDa; Table 3) exhibits a rapid, potent and long-lasting lytic activity against prominent bacterial and fungal phytopathogens (Bundó et al., 2014). Rice transgenic plants were obtained in which *cecropin A* expression was targeted to the seed endosperms by using the tissue-specific promoters *Glutelin B1* or *Glutelin B4*. Seeds from rice transgenic plants exhibited resistance to infection by fungal (*Fusarium verticillioides*) and bacterial (*Dickeya dadantii*) pathogens (Bundó et al., 2014). The mode-of-action of cecropin A in rice seeds remains to be investigated.

#### Magainin-2 (mag) and esculentin-1 (Esc28L) from frogs

Magainins are ribosomal AMPs produced in the skin of *Xenopus* frogs. Growth assays in the presence of the 23-AA magainin-2 showed that the peptide inhibited the growth of various Gram-positive and Gram-negative bacteria, a few fungi and protozoa (Zasloff, 1987). The *mag* gene encoding for the MAGAININ-2 protein was ectopically over-expressed in pearl millet (*Pennisetum glaucum*) under the control of the constitutive *CaMV 35S* gene promoter and challenged with three strains of downy mildew (*Sclerospora graminicola* viz. Sg 384, Sg 445, and Sg 492; Ramadevi et al., 2014; Table 3). While some pearl millet transgenic lines exhibited a slight decrease in disease incidence 7 days post inoculation relative to the controls, none demonstrated full resistance (Ramadevi et al., 2014). Performing statistical analyses however would have helped test the significance of these results. The authors attribute the minor response of their genetically modified plants to the complexity of cell wall and cell membrane components of the oomycete pathogen, which are similar in composition and structure to those of host plant cells. Perhaps combining MAGAININ-2 with cell wall degrading enzymes, as tested by Fogliano et al. (2002) whom combined SRE or SP25A peptides with endochitinase and/or glucanase, would prove an efficient strategy.

Esculentins are highly potent AMPs of 46 AAs exclusively found in the skin secretion of the frog *Rana esculenta*. Esc28L is a variant of esculentin-1b artificially created by substituting the Methionine residue at position 28 with a Leucine and an additional Methionine in position 47 (Ponti et al., 1999). The idea of using variant peptides to increase plant pathogen resistance while targeting them to the extracellular space was first explored by Ponti et al. (1999, 2003; Table 3). Extracellular targeting not only eliminates potential toxicity of the variant AMP toward host cells, while permitting direct contact with pathogens growing and multiplying in the extracellular space. In a follow-up study, Esc28L was fused to the Signal Peptide (SP) sequence of *Phaseolus vulgaris* EndopolyGalacturonase-Inhibiting Protein (PGIP) to target Esc28L to the secretory pathway. Transgenic tobacco (*Nicotiana tabacum*) lines were produced in which the constitutive expression of Esc28L conferred enhanced resistance to the bacterial pathogens of tobacco, *P. syringae* pv. *tabaci* and *Pseudomonas aeruginosa*, as well as against the fungal pathogen *Phytophthora nicotianae*, and moreover demonstrated insecticidal effect against drosophila (Ponti et al., 2003).

#### Cathelicidin LL-37 variant (Met37Leu) and archaic wallaby antimicrobial (WAM) peptide from mammals

The human cathelicidin antimicrobial protein hCAP18 is synthesized in neutrophils as a preproprotein which comprises a conserved cathelin prodomain, and a non-conserved C-terminal peptide. The latter is enzymatically cleaved after secretion forming LL-37, a 37-AA functional antimicrobial peptide. Inspired by the work of Ponti et al. (2003), a mutated variant of LL-37 was created by substituting the Methionine residue at position 37 with a Leucine (LL-37 Met37Leu) and was fused to the SP sequence of PGIP to elicit secretion (Jung et al., 2012; Table 3). LL-37 Met37Leu was then overexpressed in Chinese cabbage plants. Transgenic lines were subsequently inoculated with various bacterial (*P. carotovorum* subsp. *carotovorum*) and fungal (*F. oxysporum* f. sp. *lycopersici, Colletotrichum higginsianum, R. solani*) pathogens. The transgenic plants displayed enhanced pathogen resistance with decreased disease symptoms relative to the controls (Jung et al., 2012). No mode-of-action was proposed in this study but since LL-37 Met37Leu is targeted to the extracellular space of the transgenic plants, it could be assumed that a direct interaction occurs between the peptide and the pathogen.

Mammal cathelicidins as a whole are worth investigating for plant engineering programs aiming at improving pathogen resistance. Fourteen, twelve, and eight divergent cathelicidin genes were identified in the wallaby, possum and platypus genomes, respectively. Of these, the proteins WAM 1 and WAM2, and Platypus AntiMicrobial (PAM) 1 and PAM2, were tested against various bacterial and yeast pathogens, and shown to be much more potent than LL-37 (Wang et al., 2011). A phylogenetic approach was then used to design an archaic WAM predicted to have originated 59 million years ago and ancestral to the major clade of marsupial AMPs including the modern WAM1 and WAM2 (Wang et al., 2011). In theory, it should be more difficult for the modern pathogens to overcome plant resistance mediated by an archaic AMPs.

## Conclusions and perspectives

Secreted peptides with antimicrobial activities are proving useful as biocontrol agents in agriculture in order to increase crop yields by minimizing quantity and quality losses due to pathogenic diseases. Many of the peptides discussed above have been used to create transgenic plants which showed increased resistance to pathogens in *in vivo* laboratory based experiments, for example Pep1, systemin, mtk, and Met37Leu.

Recently, field trials of transgenic cotton plants expressing the tobacco (*Nicotiana alata*) peptide NaD1, which targets PIP2 in the fungal membrane in order to cause membrane permeabilization, showed increase resistance to fungal pathogens (Gaspar et al., 2014). This peptide was previously shown to have antifungal activity against a variety of filamentous fungi *in vitro* and this translated well to *in vivo* field trials using transgenic cotton plants against *F. oxysporum* f.sp. *vasinfectum* and *V. dahlia* (Lay et al., 2003a; Gaspar et al., 2014). Among the peptides reviewed here many have shown some form of inhibition of pathogenic fungi, bacteria and insects. However, in many cases this inhibition was shown *in vitro* by addition of the peptide to the culture medium or *in vivo* by spraying of plant leaves or addition of the peptide to cut petioles or in soil; however all of these approaches are small scale, laboratory based and mainly use model plants (i.e., non crop species). For the majority of these peptides no work has been conducted to test the effect of priming plants on normal growth and development in crop species. In the case of the NaD1 transgenic cotton it was noted that no detrimental agronomic properties were observed in field trials whilst in contrast transgenic potato overexpressing the DF2 defensin altered plant development (Stotz et al., 2009). It should be noted that priming could potentially negatively impact crop performance as energy may be diverted from growth and development of the plant to the defense system. However, this was not the case for the NaD1 transgenic cotton, therefore only more field trials will be able to determine if this is a genuine concern. Moreover, without incorporation of the peptide into the genome as in the case of NaD1 transgenic cotton, crops would potentially have to be sprayed with the peptide of choice as they are with fungicides and pesticides at the moment. The subsequent cost for production and application should be economically assessed in comparison with the current chemical formulation used in fields, along with the impact on the environment.

The discovery of new valuable secreted peptides is associated with the advancing technology of MS, in particular in combination with HPLC separation, which is able to yield high throughput identification and structural characterization. The approach taken by Chen et al. (2014), for identification of the CAPE1 peptide, is an efficient way to use proteomics and MS to identify novel ribosomal and nonribosomal peptides. This technique allows a comparison between different stress conditions to investigate when peptides are induced, e.g., healthy plants vs. wounded, infected etc. Not only does this approach identify peptides but it also gives sequence information for the peptides which could help with subsequent identification of the proprotein. As data-rich as LC-MS experiments can be, they usually require time-consuming, labor-intensive extraction and separation methods. Furthermore, LC-MS does not typically provide information on the localization of such compounds. Therefore, MALDI imaging technology offers a promising alternative as it not only allows the identification of AMPs but also their *in situ* tissue localization. MALDI imaging has successfully been applied *in vitro* (Debois et al., 2013). Coupled with traditional histology, MS imaging informs on cellular localization with a resolution down to 5 μm of not only proteins, and peptides like AMPs, but also metabolites such as lipids, sugars, in a multiplex fashion within the very same tissue section simultaneously (Aichler and Walch, 2015). Such MS imaging methods would be greatly advantageous if directly applied on diseased plants organs, as compounds co-localizing with visible symptoms would be potential targets for the discovery of novel AMPs.

A crucial step in validating the efficacy of AMPs toward plant disease resistance involves introducing such AMPs into the crop of interest and exposing the transformed crop to its pathogens. To our knowledge, such transgenic experiments have only been attempted with ribosomal or artificially designed AMPs. This arises from the fact that transgenic plants are produced by introducing a foreign piece of DNA which then goes through the transcription and translation machinery to synthesize the AMP. Creating transgenic plants by introducing nonribosomal AMPs, which by definition do not undergo ribosomal synthesis, remains a complete challenge. Perhaps an alternative would be to introduce the enzymes responsible for the synthesis of these nonribosomal AMPs, the so-called NRPS, into the crop in order to acquire disease resistance. Using a synthetic biology approach it has been shown that NRPSs can be genetically engineered to improve the antibacterial properties of a lipopeptide and expand its spectrum against human pathogens (Nguyen et al., 2010). We could not find any reports in the literature related to plant resistance against pathogens. However, the link between NRPSs from biocontrol agents and host crops was recently established between the mycoparasite and facultative root symbiont *Trichoderma virens* and maize plants (Mukherjee et al., 2012). The analysis of the loss-of-function mutants of *T. virens* revealed that a hybrid enzyme polyketide synthase (PKS)/NRPS, Tex13, was involved in up-regulation of the defense gene *phenylalanine ammonia-lyase* (*pal)* in maize; Tex13 was more than 40-fold induced during interactions of *T. virens* with maize roots (Mukherjee et al., 2012).

In the evolutionary arms race between plant hosts and pathogens, usually the pathogen wins due to shorter generation times which make them more dynamic than crops, thereby rapidly overcoming plant immunity. Therefore, any tactic that would give the plant host the upper hand would be of immense interest to agricultural programs. One strategy could exploit the fact that different antibiotic peptides can act in synergy, therefore introducing several AMPs in crops would broaden the spectrum of plant disease resistance. Along with the AMPs, enzymes targeting microbial cell wall could also be introduced to the crop of interest to facilitate the entry of secreted AMPs across pathogen physical barriers. Another strategy would be exploring ancient and extinct peptides as archaic AMPs might be more effective than the modern AMPs found in living creatures because microbial pathogens have not been exposed to them for millions of years. Therefore, the modern pathogen would not have developed resistance against the archaic peptide. Engineering crops expressing such archaic AMPs would not only help achieve disease resistance but also slow down becoming overcome by the targeted pathogens (Wang et al., 2011). A last promising strategy is to use *de novo*-designed synthetic peptides. It is out of the scope of this review which focuses on naturally occurring AMPs, yet special mention should be made of two recent reports in which crops transformed with genes coding for synthetic peptides displayed acquired pathogen resistance (Nadal et al., 2012; Zeitler et al., 2013).

## Funding

SB is funded by EU grant DP120103558 and PSS is funded by Australian Research Council grant FT110100698.

### Conflict of interest statement

The authors declare that the research was conducted in the absence of any commercial or financial relationships that could be construed as a potential conflict of interest.
